# Combined effects of bevacizumab with erlotinib and irradiation: a preclinical study on a head and neck cancer orthotopic model

**DOI:** 10.1038/sj.bjc.6604429

**Published:** 2008-06-24

**Authors:** A Bozec, A Sudaka, J-L Fischel, M-C Brunstein, M-C Etienne-Grimaldi, G Milano

**Affiliations:** 1 Department of Surgery, Centre Antoine-Lacassagne, Nice, France; 2 Pathology Laboratory, Centre Antoine-Lacassagne, Nice, France; 3 Oncopharmacology Laboratory, Centre Antoine-Lacassagne, Nice, France

**Keywords:** bevacizumab, erlotinib, irradiation, head and neck cancer, orthotopic

## Abstract

Clinical benefit has been demonstrated in patients with head and neck tumours receiving an anti-epidermal growth factor receptor (EGFR) agent in combination with radiotherapy (RT). Recent preclinical and clinical studies suggest beneficial effects from combining anti-angiogenic drugs with RT. To investigate the effect of combining these approaches, we evaluated *in vivo* the anti-tumour efficacy of the anti-angiogenic compound bevacizumab, a highly specific monoclonal antibody directed against the vascular endothelial growth factor (VEGF), erlotinib, an EGFR tyrosine kinase inhibitor, and irradiation given alone and in combination. Investigations were performed using a VEGF-secreting human head and neck tumour cell line, CAL33, with a high EGFR content, injected as orthotopic xenografts into the mouth floor of nude mice. Three days after tumour cell injection, bevacizumab (5 mg kg^−1^, 5 days a week, i.p.), erlotinib (100 mg kg^−1^, 5 days a week, orally) and irradiation (6 Gy, 3 days a week) were administered alone and in combination for 10 days. As compared with the control, concomitant administration of drugs produced a marked and significant supra-additive decrease in tumour mass; the addition of irradiation almost completely abolished tumour growth. The drug association markedly reduced the number of metastatic nodes and the triple combination significantly reduced the total number of pathologically positive lymph nodes as compared with controls. The RT-induced proliferation, reflected by Ki67 labelling, was reduced to control level with the triple combination. Radiotherapy induced a strong and very significant increase in tumour angiogenesis, which was no longer observed when combined with erlotinib and bevacizumab. The efficacy of the combination of bevacizumab+erlotinib and RT may be of clinical importance in the management of head and neck cancer patients.

Agents that target epidermal growth factor receptor (EGFR) potentially exert anti-tumour effects by inhibiting tumour cell proliferation and survival, as well as reducing the secretion of proangiogenic growth factors such as vascular endothelial growth factor (VEGF) and fibroblast growth factor that stimulate tumour neoangiogenesis (Perrotte *et al*, 1999; Woodburn, 1999; Ciardiello *et al*, 2001). It has been reported that EGFR targeting in tumours may also modulate the migration and formation of tube-like structures of vascular endothelial cells (Hirata *et al*, 2002). More recently, we have shown the presence of a gefitinib-sensitive functional EGFR pathway in an immortalised microvascular endothelial cell line of human origin (Bozec *et al*, 2005). Thus, in addition to direct effects on tumour cells, EGFR-targeting drugs may also impart an indirect anti-tumour effect through anti-angiogenic activity.

Several preclinical studies have examined the anti-tumour activity of inhibitors of EGFR and anti-angiogenic agents in combination and have demonstrated at least additive, if not synergistic, effects (Ciardiello *et al*, 2000; Jung *et al*, 2002; Herbst *et al*, 2003). Previous experimental studies showed potential beneficial anti-tumour effects when combining anti-angiogenic agents with radiotherapy (RT) (Wachsberger *et al*, 2005; Nieder *et al*, 2006), resulting in at least additive effects on tumour growth delay despite different radiation schedules, drugs and doses and combination regimens. Clinical research in this field is ongoing but additional preclinical studies are needed to further evaluate drug combinations, including the targeting of EGFR and VEGF signalling pathways in association with RT.

Tumour vasculature is a key target in the treatment of solid tumours, particularly in head and neck cancer (Le and Giaccia, 2003). In a recent study (Bozec *et al*, 2007), we investigated the effects of combining anti-angiogenic treatment, EGFR targeting and RT. We evaluated AZD2171, a highly potent, orally active, VEGF signalling inhibitor, gefitinib, an EGFR tyrosine kinase inhibitor, and RT. The anti-tumour efficacy of these treatments, administered alone and in combination for 2 weeks, was assessed in a VEGF-secreting human head and neck tumour cell line, CAL33, that highly expresses EGFR, established as xenografts (250 mm^3^) in nude mice. The median time to reach a tumour volume of 1000 mm^3^ was significantly increased for AZD2171 or gefitinib administered alone compared with the control. Greater inhibition of tumour growth was seen with the combination AZD2171+gefitinib compared with either drug alone, and the triple combination compared with either AZD2171+gefitinib or RT alone.

The aim of this study was to examine the anti-tumour effects of the clinically representative anti-angiogenic monoclonal antibody bevacizumab combined with the anti-EGFR tyrosine kinase inhibitor erlotinib and RT. To this end, we adopted an orthotopic head and neck cancer xenograft model consisting in injecting CAL33, a human head and neck cancer cell line, into the mouth floor of nude mice. This model was used to enable examination of the effects of treatment on the primary tumour itself and also, for a more clinically relevant condition, on disease extension to the lymph nodes in the neck. Molecular markers were also examined according to the applied treatments. Ki67 expression was considered as a relevant and classical parameter related to tumour proliferation. The key VEGF receptor present on endothelial cells is VEGFR2. In addition, the presence of VEGFR2 has been reported on tumour cells themselves, particularly in head and neck cancer (Neuchrist *et al*, 2001). For this reason, we analysed VEGFR2 expression both on endothelial cells and on tumour cells.

## Materials and methods

### Chemicals

Erlotinib was kindly provided by Roche (Neuilly sur Seine, France) and bevacizumab by our institution's pharmacy. Working solutions were prepared as follows: erlotinib (7.5 g l^−1^) was suspended in 0.9% NaCl and 0.01% Tween 80, and bevacizumab (750 mg l^−1^) was diluted in 0.9% NaCl. For both drugs, the concentrations were adjusted so as to include the daily dose in 0.2 ml of drug suspension. Dulbecco's modified Eagle's medium (DMEM), penicillin, streptomycin and glutamine were purchased from Whittaker (Verviers, Belgium). Fetal bovine serum (FBS) was obtained from Dutscher (Brumath, France).

### Cell line

CAL33, a cell line of human head and neck origin, was obtained in our institution (Centre Antoine-Lacassagne). This cell line exhibits high EGFR levels (33 794±624 fmol per mg protein; high-affinity sites determined by ligand binding assay; Dassonville *et al*, 1993) and produces VEGF (Cercina Onesto, CNRS-UMR6543, personal communication).

The cell line was maintained as monolayer culture in DMEM supplemented with 10% FBS v/v, 2 mM glutamic acid, 50 000 U l^−1^ penicillin and 80 *μ*M streptomycin in a humidified incubator (Sanyo, Japan) at 37°C in an atmosphere containing 8% CO_2_. Batches of 15 × 10^6^ cells were frozen in FBS supplemented with 5% dimethyl sulphoxide v/v in advance for injection into mice. Shortly before injection, cells were thawed and suspended in Ringer lactate.

### Mice

Six-week-old female NMRI nude mice were purchased from Janvier Laboratories (Le Genet sur Isle, France). They received, as intra-muscular orthotopic xenograft in the mouth floor as described by Myers *et al* (2002), an injection of 0.5 × 10^6^ cells suspended in 200 *μ*l of Ringer lactate (*n*=10 per treatment condition). There were five animals per cage with food and water available *ad libitum*; animals were killed and tumours collected at day 10 after cell injection by cervical disruption (as determined in a preliminary experiment, the 12th day after tumour cells injection, tumour development in the mouth floor prevented correct feeding of the animals with consequent unethical suffering). As no signs of suffering appeared during the experiment (submissive attitude, weight loss, prostration, vocalisation), no animal had to be killed before the end of the experiment. Animal experiments were performed in accordance with the regulations of our institution's ethics commission and guidelines of the United Kingdom Co-ordinating Committee on Cancer Research (Workman *et al*, 1998).

### Treatment

Three days after cell injection, the mice were treated each week with relevant vehicle (controls), erlotinib (50 mg kg^−1^, 5 days a week, 0.2 ml p.o.), bevacizumab (5 mg kg^−1^ per day, 5 days a week, 0.2 ml i.p.) and irradiation (6 Gy, 3 days a week every other day 2 h after drugs on tumour bed only) given alone or in combination; when co-administered, erlotinib and bevacizumab were given simultaneously (given the short duration of treatment − 7 days − five doses of each drug and four doses of irradiation were delivered); the doses of erlotinib and bevacizumab were chosen according to preliminary experiments (25, 50, 100 and 200 mg kg^−1^ p.o. for erlotinb and 5 mg kg^−1^ per day, 5 days a week, 0.2 ml i.p. for bevacizumab every day during 2 weeks), performed on tumour of this same cell line xenografted in the flank of animals, so that each drug given alone exerted only partial effects on tumour growth.

### Evaluation of drug effects

On the 10th day after cell injection, animals were killed and weighed. Primary tumours were measured (tumour volume was determined by the formula TV=*π*/6 × length × width^2^) and weighed, and bilateral radical neck dissection was performed (animals were killed by spinal cord dislocation and nodes and tumours were subsequently removed surgically and fixed in paraformaldehyde overnight). The effects of erlotinib, bevacizumab and RT, alone or in combination, on tumour growth were evaluated by measuring, for all groups, the mean tumour volume (MTV). Combination ratios (CR), as described previously (Prewett *et al*, 2002), were calculated from the mean tumour volumes of treated and untreated tumours at the end of the treatment period (day 10, after cell injection). This was carried out for control (MTVcontrol), treatment a (MTVa), treatment b (MTVb), treatment c (MTVc), treatment a+b (MTV a+b) and treatment a+b+c (MTVa+b+c).

CR a+b=MTVa × MTVb/MTVa+b × MTVcontrol

CR a+b+c=MTVa × MTVb × MTVc/MTVa+b+c × (MTVcontrol)^2^

If CR>1, there were supra-additive effects and if CR<1, infra-additive ones. Strictly additive effects were observed if CR=1.

The total number of pathologically positive lymph nodes, having signs of inflammation either invaded or not, having a visible diameter (greater than 1 mm), were recorded after node chain dissection after tumour removal.

### Analysis of tumour markers

All analyses of tumour markers were performed on the primary tumours, which were collected 10 days after tumour cell injection.

The expression of the microvessel marker VEGFR2 and of the proliferation marker Ki67 was evaluated using immunohistochemistry (Cell Signalling Technology rabbit monoclonal antibody 55B11 and DAKO monoclonal antibody, ref. M7240 and MIB-1, respectively). The analysis of VEGFR2 involved vessel loss and reduction in vessel area, which were evaluated both at the tumour centre and tumour periphery, and also a more diffuse labelling of tumour cells; in this case, both the intensity (1, 2 or 3) of labelling and the proportion of tumour cells were taken into account. The analysis of Ki67 took into account the proportion of labelled cells; labelling of each microscopic field was attributed to three labelling groups: group 1 with 15–50% of labelled cell nuclei; group 2 with 50–70% of labelled cell nuclei; and group 3 with more than 70% of labelled cell nuclei. For each treatment group, the relative proportion of each of the three labelling groups (1, 2 or 3) was pooled and represented as a histogram.

Forty × 10 magnification images of regions of high vascular density within the tumour were analysed to quantify both tumour angiogenesis and tumour cell diffuse labelling (VEGFR2). Whatever the studied marker (Ki67 or VEGFR2), the final score was the result of the examination of a maximum of 10, a minimum of 2 and a mean of 5 fields per tumour, which, due to the small volume of the tumour, allowed a thorough examination; 10 tumours were investigated for each treatment group.

### Statistical analyses

Comparison of primary tumour growth, node number, state of invasion and the effects of treatment on VEGFR-2 and Ki67 between different treatment groups were evaluated using the non-parametric ANOVA (Kruskal–Wallis test).

## Results

### Effects of treatments on tumour growth

**Figure 1:**
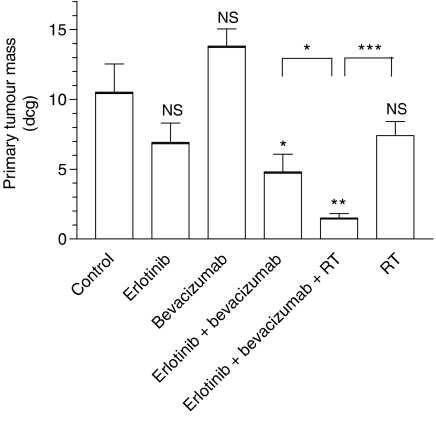
Primary tumour growth after 10 days of treatment with single agents and combinations (10 mice per treatment group). Bars denote s.d.; NS=nonsignificant (*P*>0.05); ^*^P<0.05; ^**^P<0.01; ^***^P<0.001. Values above the columns concern comparisons with the controls; other values concern comparisons between two following columns.

Treatments given alone had no significant impact on primary tumour mass, with erlotinib and RT slightly decreasing tumour growth (35 and 30% tumour mass reduction as compared to the control, respectively, *P*>0.05) (Figure 1). The combination of erlotinib and bevacizumab induced a significant primary tumour mass decrease (60%, *P*=0.028 *vs* controls) and showed supra-additive effects (CR=2).

Finally, the triple combination bevacizumab+erlotinib+RT gave the highest tumour growth inhibition (85%, *P*<0.0004 *vs* controls; *P*<0.0001 *vs* RT; *P*=0.02 *vs* bevacizumab+erlotinib). The effects of this triple combination were supra-additive (CR=2.3).

### Effects of bevacizumab, erlotinib, RT and their combinations on pathologically positive lymph nodes

**Figure 2:**
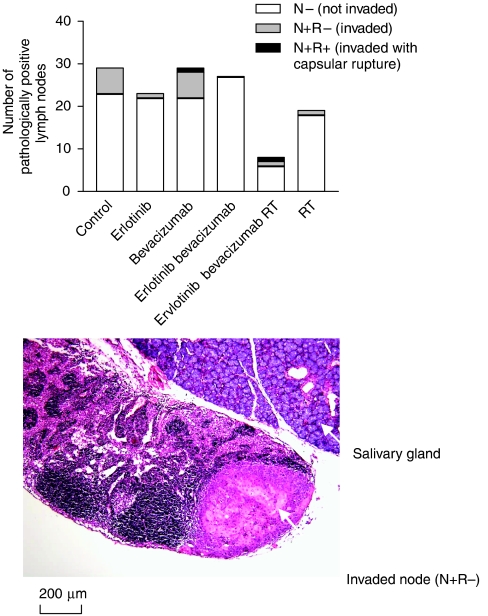
Impact of single agents and combinations on pathologically positive lymph nodes (10 mice per treatment group). The only significant differences were for node invasion status (bevacizumab+erlotinib *vs* control, *P*=0.03) and for total node number (bevacizumab+erlotinib+RT *vs* control, *P*<0.001). An illustrative example of an invaded node without capsular rupture (N+R−) is given.

The effects of single treatments by erlotinib, bevacizumab or RT on the number of nodes and the proportion of invaded nodes paralleled their impact on primary tumour mass with a slight but not significant decrease in the total node number and the proportion of invaded nodes for erlotinib and RT, and a slight but not significant enhancement of invaded nodes with bevacizumab (Figure 2).

In contrast, and corroborating the observations on the primary tumour mass, the combination erlotinib+bevacizumab totally prevented node invasion (*P*=0.03 *vs* controls). However, the drug combination had no effect on the total number of pathologically positive lymph nodes. In contrast, the bevacizumab+erlotinib+RT triple combination produced a very significant decrease in the total number of pathologically positive lymph nodes (*P*<0.001 *vs* controls) although invaded nodes were still present among these markedly reduced pathologically positive lymph nodes.

### Effects of bevacizumab, erlotinib, RT and their combinations on proliferation markers (Ki67 labelling)

**Figure 3:**
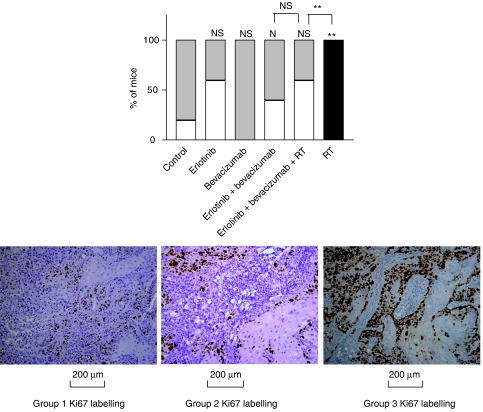
Impact of the different treatments on Ki67 proliferation marker (10 mice per treatment group). The proportion of primary tumours with labelling less than 50% is shown in white (group 1), between 50 and 70% in grey (group 2) and more than 70% in black (group 3); NS=nonsignificant (*P*>0.05); ^*^P<0.05; ^**^P<0.01; ^***^P<0.001. Values above the columns concern comparisons with the controls; other values concern comparisons between two following columns. An illustrative example of each labelling group is given.

Neither erlotinib (small decrease) nor bevacizumab (small increase) administered alone had a significant impact on tumour cell proliferation (*P*>0.05 *vs* controls) (Figure 3). In contrast, RT application induced cell proliferation (*P*=0.007 *vs* controls). This RT-related effect on tumour cell proliferation was diminished by the presence of erlotinib+bevacizumab to a level similar to the controls (*P*=0.0037 *vs* RT; *P*=0.587 *vs* controls).

### Effects of bevacizumab, erlotinib, RT and their combinations on tumour vessels (VEGFR2 labelling)

**Figure 4:**
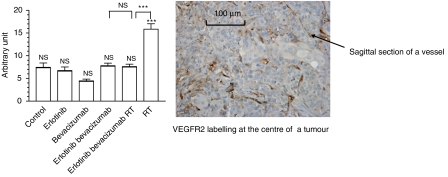
Impact of the different treatments on the number of tumour vessels labelled by the anti-VEGFR2 antibody measured at the centre. NS=nonsignificant (*P*>0.05); ^***^P<0.001. Indications above the columns concern statistical comparisons with the controls; other indications concern statistical comparisons between two following columns. An illustrative example of VEGFR2 labelling of the vessels is given.

Bevacizumab given alone induced a decrease in endothelial cell VEGFR2 labelling (Figure 4). Nevertheless, this change was not significant as compared to controls; in contrast, RT induced a strong (× 2) and very significant (*P*<0.001) increase in tumour angiogenesis. Of note, this RT-related effect on tumour vessels was no longer observed when RT was combined with erlotinib and bevacizumab. The results of the examination of peripheral tumour vessels were found to be superimposable with those of the central part of the tumour (data not shown).

### Effects of bevacizumab, erlotinib and their combination on VEGFR2 labelling of tumour cells

**Figure 5:**
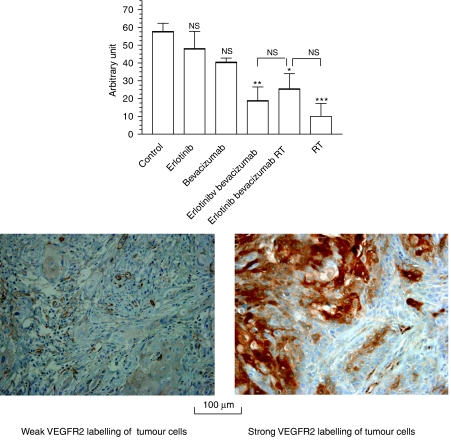
Impact of the different treatments on VEGFR2 expression on tumour cells (10 mice per treatment group). NS=nonsignificant (*P*>0.05); ^*^ *P*<0.05; ^**^P<0.01; ^***^P<0.001. Indications above the columns concern statistical comparisons with the controls; other indications concern statistical comparisons between two following columns. An illustrative example of strong and weak VEGFR2 labelling of tumour cells is given.

All targeted treatments were found to diminish VEGFR2 expression on tumour cells (Figure 5). The combination erlotinib+bevacizumab led to a significant decrease as compared to controls. Interestingly, a single application of RT alone also had a marked impact on VEGFR2-labelled cells.

## Discussion

There are recent preclinical background (Bozec *et al*, 2006) and early clinical trials (Caponigro *et al*, 2005; Meyerhardt *et al*, 2007) that indicate that targeting both EGFR pathway and tumour angiogenesis may be a useful strategy in the management of several tumour pathologies. Head and neck cancer is particularly relevant in this respect. As recently underlined by Forastiere and Burtness (2007), there are compelling clinical data showing that head and neck cancer is an ideal malignancy for EGFR inhibition either with antibodies or small-molecule tyrosine kinase inhibitors. This is especially evident for combinations between EGFR targeting drugs and cytotoxic agents such as irradiation (Bonner *et al*, 2006; Harari and Huang, 2006) or chemotherapy (Siu *et al*, 2007). This background led us to undertake the present experimental study on head and neck cancer xenograft considering the triple association of EGFR targeting by erlotinib combined with the anti-angiogenic agent bevacizumab and associated with irradiation.

Doses of individual agents were chosen to generate relatively limited effects and thus allow detection of potential interactions. This is for this reason that bevacizumab had no evident impact on tumour growth *per se* and that erlotinib exhibited moderate anti-tumour effects as a single drug (Figure 1). Interestingly, the combination of the two drugs produced supra-additive effects on the primary tumour mass with a combination ratio value at 2. We recently made a similar observation when applying on CAL33 cells growing as a classical xenograft the anti-angiogenic multi-target tyrosine kinase inhibitor AZD2171 associated with the anti-EGFR agent gefitinib (Bozec *et al*, 2007). One of the possible explanations for the presently observed supra-additive anti-tumour activity may lie in an optimal ‘vertical blockade’ of VEGF pathway not obtained by either agent alone bearing in mind that EGFR targeting leads to inhibition of VEGF tumour secretion (Byers and Heymach, 2007).

As recently underlined by O'Reilly (2006), there is a preclinical rationale to combine anti-angiogenic agents with radiation. The association between anti-EGFR therapy and radiation is based on consistent preclinical data confirmed at the clinical level, particularly in head and neck cancer (Bonner *et al*, 2006). In this study, the combination bevacizumab+erlotinib was considered as a whole when associated with irradiation. This triple combination was found to be supra-additive for cytotoxic activity on the primary tumour (CR=2.3; Figure 1). We observed both irradiation-related induction of tumour proliferation (Figure 3) and an increase in tumour angiogenesis following irradiation (Figure 4). This phenomenon may be due, at least in part, to the post-radiation induction of EGFR signalling in irradiated cells previously reported by others (Schmidt-Ullrich *et al*, 1996) and us (Bozec *et al*, 2005). It is known that VEGF secretion is under the control, at least in part, of EGFR signalling (Goldman *et al*, 1993). Interestingly, these radiation-related stimulations of both tumour cell proliferation and tumour angiogenesis were markedly diminished by the presence of the drug combination, with the EGFR-targeting drug probably playing a role in the attenuation of radiation-induced tumour proliferation. In addition, bevacizumab strengthens these latter effects by counterbalancing the local production of VEGF by the tumours. The present study confirms the presence of VEGFR2 expression in squamous cell carcinomas of the head and neck as shown before by Neuchrist *et al* (2001). Taken together, it seems likely that two overlapping systems are involved in the tumorigenicity and tumour angiogenesis with autocrine/paracrine loops using VEGF and VEGFR2. Clearly, the present data indicate that the combination of two targeted drugs with RT is particularly well suited for interrupting this vicious circle and has thus a marked preferential impact on tumour cells carrying VEGFR2. The presence of expression of VEGFR2 in head and neck cancers could be indicative of cases potentially sensitive to this innovative combination.

This study was also specifically designed to examine the impact of treatment not only on the primary tumour itself but also on the local metastatic invasion in nodes, thus creating a condition of clinical relevance. This was made possible by the adoption of a head and neck orthotopic model (Myers *et al*, 2002). This aspect is particularly relevant for head and neck cancer, as this type of cancer has a loco-regional evolution with frequent neck involvement, which is the most important parameter for prognosis (Pentenero *et al*, 2005). Of importance, the present data demonstrate a differential effect of the respective treatments on node involvement, lending additional clinical significance to the observations obtained from the examination of the primary tumours. Thus, the combination erlotinib+bevacizumab confirmed the supra-additive anti-tumour effects by markedly reducing pathologically positive lymph nodes whereas the activity of single drugs at this level was less evident, particularly in the case of bevacizumab (Figure 2). Of clinical interest was the fact that the therapeutic condition leading to fewer macroscopically evident nodes was the triple combination erlotinib+bevacizumab+irradiation.

In total, this study provides another strong rationale for a combination between irradiation and EGFR pathway targeting plus anti-angiogenic treatment (Bozec *et al*, 2007). The conclusion drawn from the specific experimental conditions with a head and neck cancer orthotopic xenograft model strengthens the clinical applicability of the presently investigated treatment strategy not only for the management of head and neck cancer but also for other major tumour pathologies such as non-small-cell lung cancer (Maione *et al*, 2006), colorectal cancer (Arsene *et al*, 2006) and glioblastoma multiforme (Newton, 2007) in which EGFR targeting and anti-angiogenic treatments are particularly active.
